# Fermenting value on Vietnamese coffee farms: working knowledge and the production of quality

**DOI:** 10.1007/s10460-025-10823-z

**Published:** 2025-12-17

**Authors:** Skylar Lindsay

**Affiliations:** School of Geographical Sciences, University of Bristol, University Road, Bristol, BS8 1SS UK

**Keywords:** Knowledge systems, Commodification, Production networks, Categories, Global value chains, Cultural economy

## Abstract

Making a living in commodity production depends on knowledge of both *how* to produce and *what* is produced—a commodity’s qualities and value. Producers’ livelihoods and agency stem from the relationships between their knowledge systems and value chains. Bringing together agrarian studies with cultural-economic studies of value chains, this paper considers knowledge(s) as forces in the production of diverse kinds of value, complicating labels like expert, technical, lay or local. The paper presents the case of specialty coffee farming and processing in Vietnam, focusing on how fermentation during post-harvest processing creates qualities and value. The analysis shows coffee producers employ *working knowledge*: knowledge of production that *does* work by producing value. Though Vietnam is the world’s second-largest coffee producer, it has a reputation for poor quality. In the 2010s, a slim minority began producing specialty coffee—valued for exceptional flavors and aromas—in part by employing novel post-harvest fermentations. Through an ethnography of fermentation knowledges and practices, this paper shows that livelihoods depend on flows not only of coffee and its material qualities but also of information. Producers assemble and translate diverse knowledges and practices as their work depends on making a commodity legible for value chains. Their knowledge systems are thus not peripheral but central to global production, as they shape and are shaped by value chains and the production of quality.

## Introduction

At 6 AM in Vietnam’s Lâm Đồng province, mist still rises from the road as Huy^1^, a 48-year-old coffee farmer and processor, and I sit watching steam from our cups of strong black coffee. We talk about picking, pulping and fermentation. All coffee fruit ferments after picking but particular methods can alter and develop flavors and aromas, helping set Huy’s coffee apart from the coffee in our cups at this roadside cafe.

Huy’s ageing father drives up, parks his motorbike and sits down with us. The two of them cultivate a three-hectare farm and run a mill, hiring 2–10 workers to help during harvest. Their labor and knowledge produce coffee that inhabits and shapes distinct commodity categories: sold to roasters in Vietnam and abroad, it fetches around twice the price of commercial-grade coffee—a rare but potentially transformative premium. Huy’s father was a rice farmer in central Vietnam but now supports his son in navigating the prospects and contingencies of quality and value amid a volatile, inequitable industry. For Huy, his father and the workers, knowledge of work and the commodity at hand has shifted the contours of their livelihoods, experiences of coffee production and their place within value chains.

This paper begins from a provocation that all commodity production can be studied as knowledge work (Blackler 1995; Jessop 2005), as all workers sell their knowledge as expressed through labor. Producers’ knowledge is often central to both political ecology and agrarian studies (van der Ploeg 1993; Bentley 2006; Carolan 2006; Krzywoszynska 2016; Huynh et al. 2022; Peddi et al. 2023), however most studies focus less on how producer knowledges work within and shape commodity flows. This paper extends their analysis by bringing it together with cultural economic approaches to value chains (Appadurai 1986; Callon et al. 2002; Bair 2009; Besky 2020; Turner et al. 2022); it argues, through a case of coffee farming and processing in Vietnam, that the agencies and roles of producers are shaped by the relations between their knowledges and global value chains. Following knowledge through value chains introduces new understandings of the work and agencies of commodity producers.

A livelihood of making is contingent on both *know-how* and also *know-what*: labor deploys both capacity to produce and knowledge of what is produced. From factories and workshops to farms and mills, actors communicate and impose requirements and practices, contributing to the construction of categories that classify a commodity as one thing or the next (Stanford 2002; McDonell 2023). Dynamics of distribution, power and governance in value chains (Gereffi et al. 2005; Dallas et al. 2019) are thus shaped not just by macro-level forces but also by knowledge systems: by what counts as knowledge, how it is learned and how it evolves (Šūmane et al. 2018; Whitt 2009 in Liboiron 2021). Power shapes the translation of knowledge and materialities into commodity categories. The argument I advance analyses the *function* of knowledge systems in relation to value and quality.

This paper analyzes how specialty coffee producers in Vietnam employ practices of fermentation in post-harvest processing as well as information about these practices to create value and quality. “Producers” refers to people who farm or process coffee. Over 90% of processors in this research were also involved in farming. With a few exceptions, specialty coffee processing in Vietnam happens in small mills on farms rather than in factories. While all producers hired workers, at least for harvest, this paper focuses on the knowledge and interactions of producers.

Coffee ferments as sugars in fresh coffee fruit (cherries) meet bacteria and yeasts in the environment after picking. Fermentation is both a mechanical tool that helps remove sticky mucilage from beans and a catalyst for quality that shapes beans’ chemical makeup, impacting the coffee’s sensory qualities. Microbial activity thus presents risk, via molds and spoilage, and also opportunities to create different types of value.

Cultural-economic studies of commodities (Callon et al. 2002; Lind and Barham 2004; Besky 2020) show that in both production and consumption, quality and value are always also plural: a commodity’s goodness and characteristics (qualities) and its diverse worths (Graeber 2001; Sayer 2011; Grant 2022; Akram-Lodhi et al. 2025) to people and beyond are relationally constituted—produced in physio-cultural processes.

Though Vietnam is the world’s second-largest coffee producer, historical and ongoing processes of quality construction have given it a reputation for poor quality coffee. This is partially because 96% of production in Vietnam is Robusta coffee (Nguyen 2025)—a less valued and often lower quality relative of more popular Arabica^2^. However in the mid-2010s, a small minority in Vietnam began producing specialty-grade Arabica and Robusta with highly distinctive sensory (material) attributes, in part through careful fermentation.

Methodologically, the research is a multi-sited ethnography of the knowledges and practices of farmers and processors, nearly all of them smallholders with less than 5 ha of coffee. I learned from coffee producers and hired workers by working with them and learning-by-doing (McMorran 2012), adopting an ethnographic approach driven by the material elements of livelihoods (Woodward 2020): coffee trees, other plants, machinery, fruit, bacteria, yeasts, tools, soil and more.

I describe how producers enact *working knowledge*: this is knowledge of *how* to work, produced *through* work; it is also “working” in that it is evolving and good enough for producers’ needs. While these descriptions echo existing analyses in political ecology and agrarian studies (van der Ploeg 1993; Šūmane et al. 2018; Peddi et al. 2023), this paper offers a novel addition by showing how producer knowledge *does* work in that it contributes to the production of value—both exchange value and symbolic value for producers’ identities and broader cultural economies.

As producers communicate how a commodity was produced, they communicate *what* it is, engaging in power-laden processes of category construction (McDonell 2023; Zinsli 2023). By analyzing the function of producer knowledges in value chains, a working knowledge framework complicates labels like expert, lay, local and traditional; knowledges are always multiple, troubling categories. In practice, knowledge both disrupts its own classification and drives the classification of commodities.

In this case, coffee processors engage in two acts of translation (Callon 1984; see also Star and Griesemer 1989; Barham 2016). First, they translate diverse knowledges, including from social sharing, formal trainings, written sources and direct experience, to form their own working knowledge of production. Deviating from common, lead firm- or state-backed practices in Vietnam, which focus on quantity and service mass market demand, they engage in what might be called a “political agronomy from below” (see Sumberg et al. 2013; Taylor et al. 2021): they critically assess diverse knowledges of production and consider the implications of each practice or principle for their coffee and their livelihoods. Second, as processors sell their coffee, they translate information about production in order to make the commodity legible in the value chain. Their knowledge classifies and qualifies the coffee, helping to shape categories and produce value (Stanford 2002; McDonell 2023; Tunarosa 2023; Zinsli 2023). The capacity of producers’ knowledge systems to shape constructed categories illustrates their power and agency, or lack thereof, within a value chain.

**Fig. 1:**
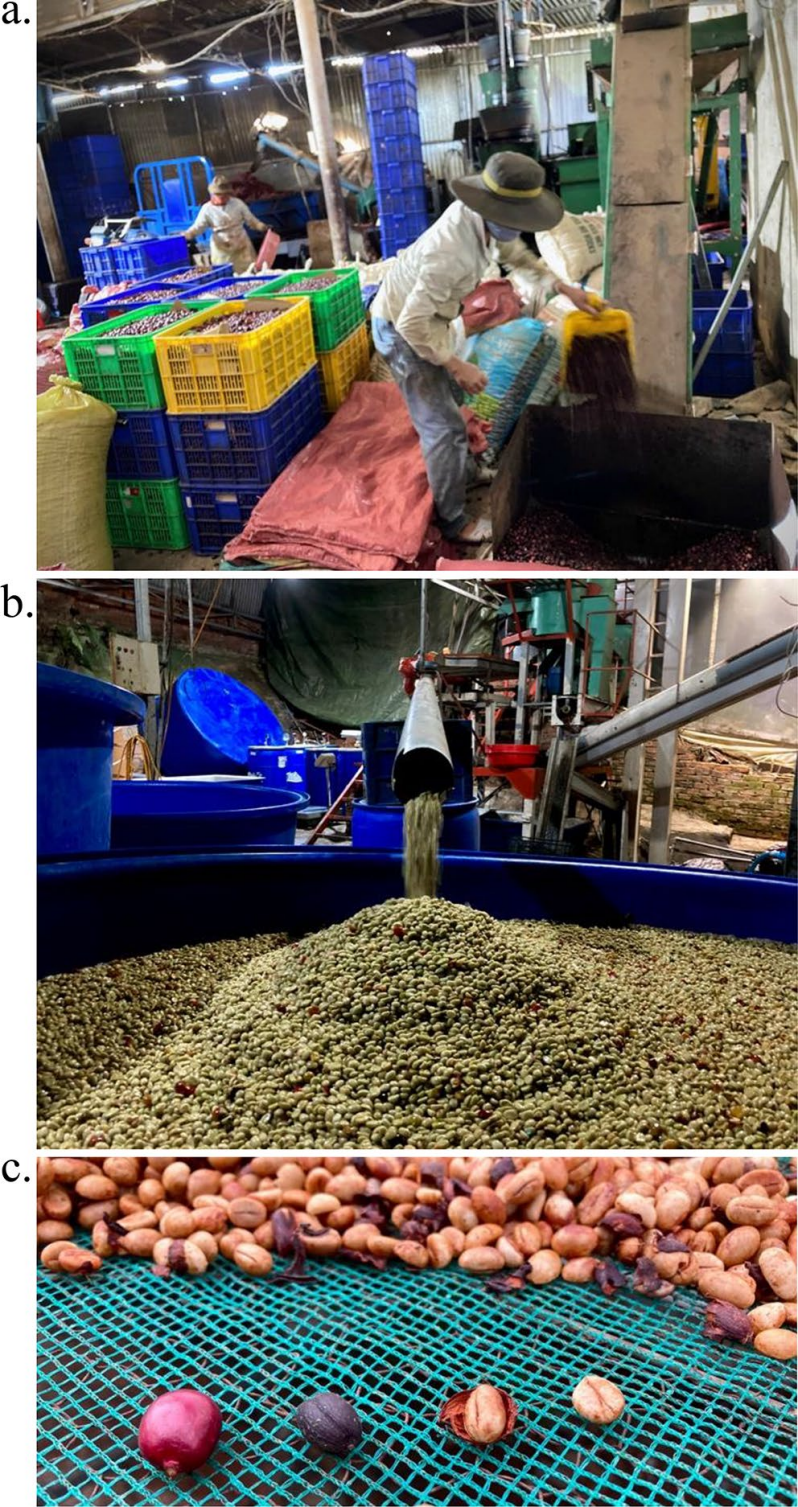
Coffee processing: (**a**) Coffee cherries go into a mill; (**b**) pulped coffee for submerged fermentation; (**c**, left-to-right) fresh cherry, natural-processed coffee (dried whole), honey-processed coffee (dried with mucilage), washed-processed coffee (pulped, soaked to remove mucilage via fermentation, washed, then dried)

People selling specialty coffee differentiate products based on specifics of production such as precise origin (specific farm or mill), variety of coffee tree (Bourbon, Geisha, etc.) and processing method—variations on washed, natural, honey, etc. (Fig. 1; also Feran 2022). Processing methods are distinguished in part by how coffee ferments: it may be dried as whole cherries, pulped and soaked in water, packed in sealed containers or accompanied by fruit to co-ferment. Huy and his father sell coffee by communicating specific understandings of what happens on the farm and in the mill. Buyers and downstream actors articulate specifications and standards but quality and value are realized through that transfer of knowledge. Commodification is thus a physio-cultural translation process, drawing together the labors of microbes and their metabolites, sensory perception, socially-embedded markets and politics of expertise and connoisseurship.

**Fig. 2:**
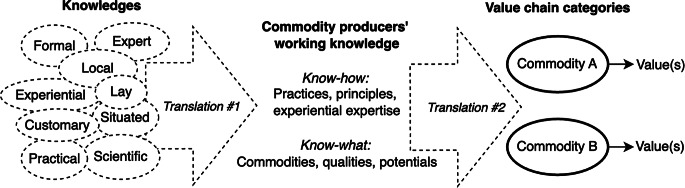
Functions of commodity producer knowledge in value chains

**Fig. 3:**
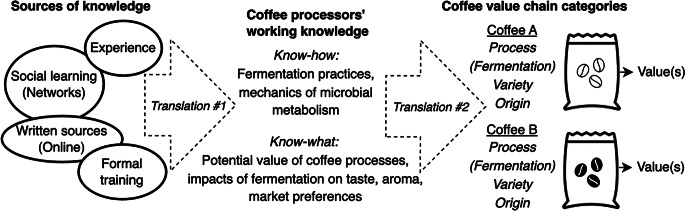
Coffee processor knowledge of fermentation in value chains

The following sections begin with discussions first of political ecology and agrarian studies approaches to knowledge and then of other existing invocations of the term “working knowledge”. I then describe a material-driven ethnographic methodology and outline the historical materialisms of coffee quality. I then discuss processors’ specific fermentation practices and how processors build knowledge of fermentation, including through experiential, social and formal learning (Figs. 2 and 3). I show how producer knowledge is crucial to realization of value and quality as it allows the construction of commodity categories, analyzing the specific implications for how agrarian studies, cultural economics and studies of value chains might approach livelihoods of commodity production differently.

### Political ecologies of knowledge

To think through producers’ knowledge, this paper expands on understandings from political ecology and agrarian studies (Natarajan et al. 2022; Peddi et al. 2023; Zinsli 2023; Hajdu et al. 2024; Akram-Lodhi et al. 2025) by bringing in cultural-economic approaches to value chains (Callon et al. 2002; Bernstein and Campling 2006; Bair 2009; Krzywoszynska 2015; Besky 2020).

Knowledge always exists evolving systems, in which different knowledges “count” differently and power dynamics are critical (Whitt 2009, 31–38; see Liboiron 2021). Whitt writes specifically on conflicts and consequences between and among indigenous and dominant knowledge systems (Whitt 2009, 31–38). This framework helps in studying how knowledge flows through uneven production networks, revealing where and how it impacts the production of value and quality.

Agrarian studies have long emphasized how dynamics among different knowledge systems are essential to understanding livelihoods as embedded in place and ecology (van der Ploeg 1993; Scoones 2009, 183–185). Such studies consider how producer knowledge is situated and contextual (Bentley 2006), practical (van der Ploeg 1993), experiential (Krzywoszynska 2016), and localized (Šūmane et al. 2018; M. P. Nguyen et al. 2020; Huynh et al. 2022). These framings show producer knowledge is necessarily political, marked by knowledge hierarchies (Scoones 2009) and divides of local/generalizable and lay/expert (Carolan 2006). Critiques of rural development studies have suggested researchers and practitioners marginalize some knowledges while granting power to others, influencing portrayals of agrarian lives (Natarajan et al. 2022). These approaches each offer particular characterizations of peasant knowledge, or *savoir-faire paysan*—knowledge as practiced in agrarian places and lives (Lacroix 1981 in van der Ploeg 1993).

These characterizations reflect the Vietnamese term thành thạo, which refers to practical expertise built through extensive experience. Farmers and agricultural engineers (*n* = 4) invoked thành thạo in interviews to describe coffee producers with locally-applicable expertise, in contrast to more formally or institutionally-recognized expertise—chuyên gia. In Sarah Grant’s anthropology of coffee and risk in Vietnam, she tells how farmers used thành thạo to describe experiential knowledge built through work (2014, 71). Grant argues that “on a local level, multiple ideas about what constitutes legitimate knowledge and expertise challenge norms about how coffee should be produced, traded, and consumed” (2014, 72). Like the framings above, this analysis is essential as it points to how an assemblage of local expertise is a source of bottom-up momentum in farming knowledge systems—a political agronomy from below (Sumberg et al. 2013).

Research in organizational studies and science and technology studies has also invoked a concept of working knowledge to describe how everyday local working practices constitute regimes of knowledge (Harper 1987; Davenport and Prusak 1998; Berner and Björkman 2017). Douglas Harper was among the first to develop the idea, in a portrait of a mechanic and the community surrounding his garage in northern New York (Harper 1987). Frank Blackler applies a concept of working knowledge in an often-cited critique on knowledge work (1995), arguing that knowledge may be embrained (grounded in cognitive skill), embodied (physically known), encultured (achieved through shared understanding), embedded (engrained in systems and place) and encoded (represented). Through these processes, everyday practices shape which types of knowledge and learning become valued.

This paper’s framework analyzes the *functions* of knowledges (Fig. 2) by reading them through lenses from cultural economy. This literature studies the role of quality in value chains, through analyses of standards (Stanford 2002; McDonell 2023), conventions (Ponte 2016; Fischer 2024), governance (Gereffi et al. 2005; Dallas et al. 2019) and quality itself (Hébert 2010; Besky 2020).

Research on coffee value chains has shown how social-cultural factors impact distribution and production of value (Daviron and Ponte 2005; Vicol et al. 2018; Fischer 2022) and of power, authority and expertise (Grabs and Ponte 2019; Quiñones-Ruiz 2020). Studies of agronomic change in Vietnam have also analyzed the construction and impacts of knowledges and expertise (McElwee 2016; Huynh et al. 2022), including for coffee farming in particular (M. P. Nguyen et al. 2020; Grant 2023).

However studies of value chains, as well as cultural economics generally, have rarely elaborated on *how* producer knowledge plays a role in the creation of value and quality. This paper brings these approaches together to trace how agrarian producers’ working knowledge contributes to both the recognition and realization of value and quality (Fig. 2).

## Methodology

The arguments here reflect 12 months of research with some of the first farmers and processors in Vietnam to produce specialty coffee. Methodologically, this is a material-driven ethnography of work and knowledge (Woodward 2020): I seek to understand people’s labor by focusing on the materials they work with—in this case trees, cherries, fermentation tanks, microorganisms, their aromatic metabolites and much more. I use interactions between people and these things to unpack contextual political-ecological relations. This approach reflects methodologies in sociologies and geographies of materials and labor, such as what Sophie Woodward calls “thinking with things” (2020) or what Chris McMorran, in a study of labor in Japanese inns, terms “working participant observation” (2012).

This on-the-farm and in-the-mill research focused on farmers and processors in Vietnam’s Central Highlands, an area of four provinces that produces 90% of Vietnam’s coffee. Before provincial boundaries were redrawn in 2025, coffee accounted for 30% of the Central Highlands’ GDP (ICO 2019). Through consultation with my official hosts at the Western Highlands Agriculture and Forestry Science Institute (WASI), I worked primarily in Lâm Đồng province, as political and administrative dynamics there allowed me to conduct research independently through relationships with producers. The province is also unique because its varied elevations accommodate both Arabica and Robusta cultivation.

I first contacted specialty roasters and producers in Vietnam via social media, using purposeful, illustrative sampling (Manzo 2010). After visits with some, I asked their help in meeting other producers well-positioned to speak to the development of Vietnamese specialty coffee. The specialty industry of producers and domestic roasters is small. Most have close business and personal ties and are happy to make introductions. By visiting many farms and mills and asking about people’s practices, networks and the specialty industry’s brief history, I situated my new contacts and teachers within the industry landscape.

I made repeated visits to two producer networks in Lâm Đồng, one Arabica and one Robusta, because they produce some of Vietnam’s highest quality coffee (according to roasters and graders) and have helped the industry grow. They and their hired workers also permitted me to work alongside and learn from them.

My methods involved participating in coffee farming and processing: planting trees, digging holes, fertilizing, picking cherries, transporting them, sorting, washing, pulping and eating them (a check of ripeness and sugar content). “Research” also meant packing coffee into fermentation tanks, checking and stirring coffee as it ferments, cleaning processing vessels and machinery, raking drying coffee, and eventually following processors as they sold their coffee down the value chain. These go-alongs included public events, coffee cuppings (orchestrated tastings) and visits with potential buyers. I also accompanied roasters and state, private and civil society agricultural experts on farm visits.

I also conducted semi-structured interviews with 54 specialty coffee producers, most of whom both farm and process coffee, 15 commercial-grade farmers and 28 key informants: 6 agricultural researchers or engineers, 9 Vietnamese roasters, 4 overseas roasters, 5 Vietnamese exporters and 4 overseas importers. Many key informants let me join them to visit farms and mills. Thirty-three interviews were conducted with one of two translators (themselves coffee producers) in Vietnamese; I conducted the remainder independently in Vietnamese as my language skills improved, except for interviews in English.

This methodology—working and then asking about it—allows in-depth study of producers’ working knowledge (Harper 1987, p. 53). A parallel appears here between this ethnography of practices and knowledge and producers’ own experiential, embodied learning, as their livelihood decisions depend on knowledge built on and with the ground. My research gleanings are a fraction of their expertise, but they allowed me insight into the struggles, messiness and friction of production (Tsing 2008)—the struggles of fermenting coffee and making quality.

### Histories of coffee qualities

Vietnam stands out among coffee producing countries for four things: its rapid rise from minor producer to second-largest globally in under 20 years (1986–2000), a poor reputation for quality, a reliance on Robusta and exceptionally high yields-per-area (averaging 2.3 tonnes/ha, partially due to the reliance on Robusta; ICO 2019). Landholdings are also smaller than in many coffee-producing countries and households often have multiple plots. Household farms constitute 85% of coffee by area and 63% are under 1 ha (ICO 2019).

Until the mid-2010s, almost no Vietnamese coffee qualified as specialty-grade. “Specialty” is often defined under the protocols of the international^3^ Specialty Coffee Association (SCA) as coffee scoring at least 80/100 on a sensory evaluation scale when cupped (tasted) by a certified Q Grader. Q Graders must pass an exam with the Coffee Quality Institute (CQI) and follow metrics and protocol written by the SCA Standards Development Panel^4^. Higher-scoring specialty coffees can sell for multiple times the price of undifferentiated coffee, often called “commercial” or commodity coffee.

Like many categories, specialty coffee has a material basis but is culturally-constructed (see Liberman 2022 on grading, tasting and their phenomenologies). This coffee is sold by quality-focused roasteries and cafés globally, with cultural capital and the largest markets concentrated in Europe, North America, Australia, Japan and Korea. Roasteries and cafes in the Global South follow similar aesthetics and norms but also adapt and transform them; in the case of Vietnam, domestic preferences for strong Robusta brewed with a *phin* filter persist and mix with growing interest in specialty coffee.

The specialty subsector in Vietnam is so small and rapidly evolving that there are no systematic estimates of its size. National coffee competitions for producers, such as Vietnam Amazing Cup, report larger volumes scoring over 80 points on the SCA scale every year. Relative to national output, this is still the less-than-1% of coffee. Vietnam’s specialty coffee subsector began in the early 2010s with a few enterprising Arabica farmer-processors in Lâm Đồng who had the necessary capital and connections to domestic roasters interested in supporting them. Lâm Đồng’s Arabica benefited from foreign and domestic investment and had already gained a positive reputation in Vietnam for cup quality. A few producers and their supporters built on this success and became the first in Vietnam to produce specialty coffee, however they operated as though only Arabica could be differentiated based on desirable tastes and aromas. The concept slowly spread to Robusta producers between 2015 and 2020, aided by CQI’s introduction of its first specialty (“fine”) Robusta program in 2010 (developed with the Uganda Coffee Development Authority; see Hetzel 2015).

The global coffee trade’s architectures of classification, built first by colonial commerce and again by Global North institutions and businesses, have used categories of Arabica as the definitional types for gradations of high quality. These architectures cast Robusta as cheap and poor quality and in a self-fulfilling prophecy, Robusta was relegated to the race-to-the-bottom market for instant coffee, cheap blends and caffeine extract (Frith 2022; Pociecha 2022).

Robusta is often intense, chocolatey and nutty and can taste like warm spices and rich dried fruits. If picked unripe or processed or brewed poorly, it can be overly bitter, woody or earthy (World Coffee Research 2023). A small group of specialty Robusta advocates in Vietnam, Uganda, India and elsewhere have begun to change both its qualities and reputation.

Vietnam came to rely on Robusta in the decades following the 1986 Đổi Mới economic reforms because it offered high yields and resistance to leaf rust, filled the criteria of socialist bloc trading partners (chiefly East Germany) and grew well in the climate of Vietnam’s Central Highlands (Dang and Shively 2008; Schwenkel 2022).

Vietnam’s Arabica is also not known for being high quality. This is partially because Vietnam mostly grows Catimor, an Arabica variety bred from a spontaneous Arabica-Robusta hybrid (Hybrido de Timor), which often has a limited cup profile and commands little value in specialty markets. Though Arabica is a small percentage of Vietnam’s production, this still amounts to far more than well-known Arabica origins like Kenya and El Salvador.

But coffee science^5^ does not attribute quality to a single element of production. Coffee of a single species, origin or processing method does not inherently taste a given way. Instead, quality is shaped by interactions between elements in production (human and non-). Fermentation during post-harvest processing—the steps that turn fresh cherries into dried “green” beans to be shipped—is critical to changing coffee quality (Brando and Brando 2014; Pereira et al. 2021). This is especially true for both Robusta and Catimor, as fermentation can develop more widely-desired qualities and reduce less-desired notes (“defects”). Fermentation has the potential to, as coffee processing specialist Solis (2020) puts it, “democratize quality” by changing what is possible.

Coffee quality categories and conventions are both highly entrenched and also hybrid. Specialty coffee conventions for quality are tied to systems of knowledge established by companies and organizations (SCA and CQI) in the Global North, often dominated by White men. These architectures of quality are primarily enacted and maintained in spaces, such as SCA Expo, World of Coffee and World Barista Championships, that are often inaccessible to producers due to costs and visas. When people from coffee producing countries are able to affect legible commodity categories, they do so by working within such structures. The hybridity of Vietnam’s specialty coffee industry, enabled by rising middle class incomes, breeds new possibilities for value chains and value capture.

Producers’ success in differentiating their coffee is highly dependent on the relationships between their knowledge systems and these value chains—on how and what they learn of fermentation and other practices, and on how and what they communicate to their value chains and networks.

### The work of fermentation

This section begins by outlining how fermentation impacts cup quality and then describes how processors practice fermentation and the flows of fermentation knowledge. All processing involves fermentation, as attendant microbes from the environment find the sugars of fruit flesh. However the forms and functions of fermentation vary, from tool, to source of risk to catalyst for qualities. Processes differ primarily in how much fruit flesh and underlying mucilage (mesocarp) is removed before drying and how it is removed: mechanically through milling and/or through fermentation. Whether coffee is pulped and submerged in water or dried as whole cherries, microorganisms consume sugars in mucilage and the sticky layer loosens, to then be washed away or hulled in dry milling (see Fig. 1).

Fermentation presents significant risk to producers as harmful fungi may produce compounds such as ochratoxin A, which at high levels make coffee unfit for export and toxic to consume. As Vietnam’s coffee industry has boomed, the biopolitics of these toxins threaten livelihoods in the value chain and the place of Vietnamese coffee in the global trade (Grant 2014, 2023).

However fermentation also develops particular chemical compounds in the coffee bean which transform into flavor and aroma molecules through roasting: pressure increases, cell walls break down, chlorogenic acids, polysaccharides and proteins degrade, amino acids and sugars condense and caramelize (Cardoso et al. 2021). Roasted coffee’s sensory characteristics are the result of around 800 volatile compounds and many more non-volatiles (Pinheiro et al. 2021). Many of these flavor and aroma precursors are bacterial and yeast metabolites: products of life amid the coffee (Silva 2014; Haile and Kang 2019). Through these biochemical relations of on-farm and in-mill ecosystems, coffee producers intervene in the material production of quality, shaping and contesting value (Tsing 2008) through their knowledge of materials and their possibilities.

Coffee fermented in a specific way can become a new category of commodity (Tunarosa 2023). The common processing labels “washed” (dried without mucilage), “honey” (dried with mucilage) and “natural” (dried as whole cherries) involve different fermentations. When processors adopt specific protocols, they can further differentiate coffee; Vietnamese Robusta becomes, for example, “72-hour anoxic honey processed”. Though this alone tells consumers little about its possible sensory qualities (tastes, aromas, body), it turns undifferentiated coffee into “a coffee”: a distinct lot that undergoes its own process of categorization.

The work of making any commodity category requires knowledge of both the physical and cultural production of value and quality. Demarking categories involves two acts of translation (Callon 1984): first, processors assemble and think through diverse knowledges—ways of knowing and harnessing materials and their possibilities for quality and value (Fig. 3, left side). The synthesized, applied expertise that results is a working knowledge, enabling a political agronomy from below (Fig. 3, center). While this discussion focuses on processors, these principles apply also to farmers and others who “make” for a living.

In a second act of translation, processors also communicate to other actors about the provenance and process behind the coffee, making claims that drive the realization of quality and value (Fig. 3, right side). This analysis offers key insights into both how knowledge *does work* in the value chain, as a commodity is categorized and valued (Stanford 2002; McDonell 2023; Tunarosa 2023), and into how an individual’s agency and power in the value chain are related to flows of knowledge. The following sections describe the actual work involved in fermentation, producers’ sources of knowledge and the role this knowledge plays in shaping value chains.

#### Practicing fermentation

At 7 AM on a small road outside an agricultural town in Lâm Đồng province, Tiến’s coffee mill is up and running. Through careful washing, milling, fermentation, drying and storage, his operation makes some of Vietnam’s highest quality coffee.

Tiến guides a small truck stacked high with plastic crates of red, ripe Robusta cherries as it backs into the small concrete and corrugated tin warehouse and begins unloading with the help of one of the workers. The crates land beside dozens of reused fertilizer bags full of cherries, all harvested the day before on one of the 30-odd farms that supply Tiến with fully ripe cherries—the essential raw material for producing specialty coffee. Tiến has his own 1.5 ha farm but his mill produces around 40 tons annually, requiring approximately 180 tons of cherries, and he spends many days making sure the other farmers understand their role in specialty coffee: in addition to carefully harvesting ripe cherries (or hiring workers to), Tiến asks that farmers reduce chemical inputs, as he says (like many farmers in this research) that excessive synthetic fertilizers and pesticides reduce the coffee’s quality. He also helps them to access the same organic fertilizers he uses. Tiến typically pays farmers 50–100% above the commercial-grade price, more than enough to cover the extra costs of paying pickers to select only ripe cherries.

Four workers, Tiến and I clean and prepare the cherries for fermentation, guiding them through five machines: bucket elevator, cleaning table to shake off debris, washer, pulper and separator. The workers here travel over 600 km from central Vietnam as part of a larger wave of seasonal migration, typically returning for multiple years. Despite coffee’s instability, it is still a lucrative option for seasonal workers. Specialty coffee also offers three-months’ work on a single farm, compared to one month for commercial-grade coffee, because farmers wait for cherries to fully ripen and harvesting is slower.

Tiến looks towards me at the end of the line occasionally to see how I’m doing: I take baskets of pulped beans as they come from the separator, dripping wet, and pour them into the top of a three-meter tall, cylindrical steel fermentation tank. The tank of cherries is then sealed and, weighing as much as a ton, wheeled into an air-conditioned room. After three days, Tiến and one worker pull it out, hoist it on a homemade pully system and tilt it nearly upside down. They coax the coffee out with a long stick and the beans come out sticky, mucilage clinging to the final endocarp layer. The smell is musty and thick, like mashed stone fruit and raisins. As beans come out, two workers and I fill plastic crates and carry the coffee to dry on mesh beds in open-sided greenhouses for 10–15 days.

Tiến has taught the workers and I the mechanics and particularities of fermentation, specifying how to handle each truckload of cherries and what they will become in the market. This batch of coffee (or “lot”) is honey-processed, however it is the extended fermentation that sets the coffee apart into a separate category altogether.

To learn practices like these, Tiến and other processors bring together heterogenous knowledges from: (1) direct experience in both mills and markets; (2) social sharing among peers and other industry actors; (3) secondhand written sources; and (4) formal trainings (Fig. 3). The assembled ways of knowing overrun single labels such as expert, lay, local and traditional and the spaces in between. Instead, specialty coffee processors, like other commodity producers, translate information (Callon 1984, p. 198) and adapt it to their materials, needs and context. Over time, their labor generates situated, applied expertise—this is *working knowledge*.

#### Experiencing fermentation

**Fig. 4:**
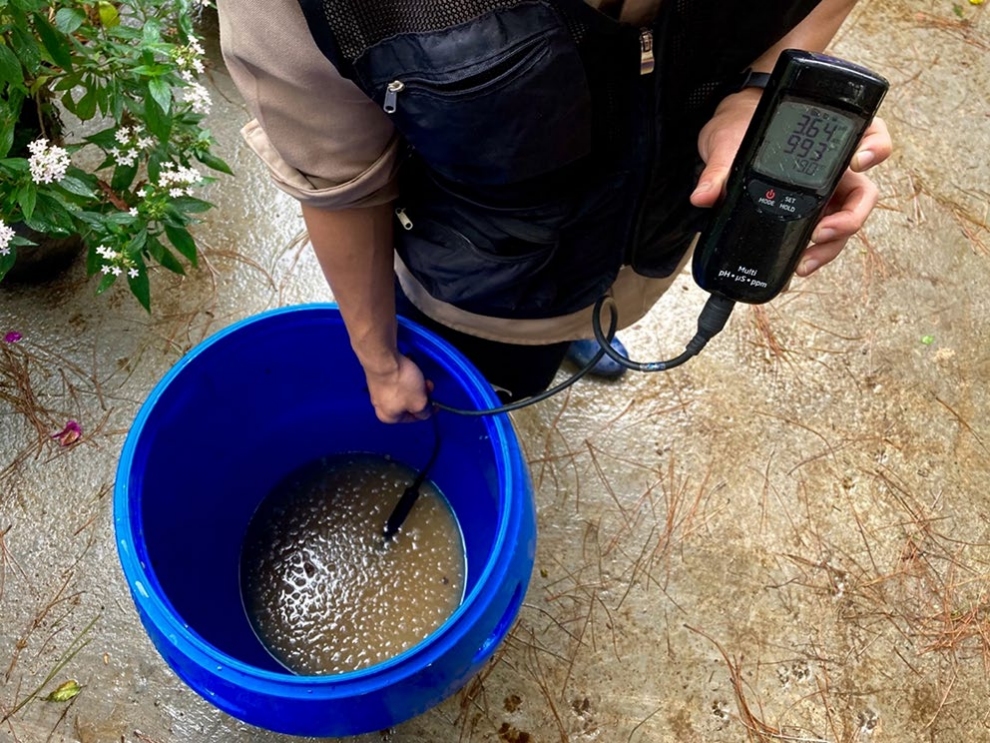
A processor checks the pH of a test batch of fermenting coffee by digital meter

As processors begin to produce specialty coffee, they build a novel kind of experiential expertise, more thành thạo (practical, built over time) than chuyên gia (formal). They conduct fermentation trials, collecting data like pH, temperature, sugar content or in rare cases microbial activity as the coffee is fermenting. Processors then manage these variables to shape ecosystems of yeast and bacteria, slowing or quickening fermentations and influencing the character of the resulting coffee. For example, they may ferment coffee in warmer or cooler spaces or add sources of sugars and microbes. These could be purchased yeasts for inoculation (bacteria are less common and more expensive), cultured “wild” yeasts or fruit. Processors often also monitor pH using a digital meter (Fig. 4). When pH drops to 4 or 5, they rinse the coffee to interrupt fermentation and drying begins. Extended fermentations also allow processors to delay drying until rain passes, a common issue when harvest begins in December, or until space opens up on mesh beds or patios. This is especially important as rainfall patterns grow increasingly unpredictable.

Experiential expertise allows producers to better manage fermentation. As Tiến puts it, “Everything is fully controlled, so the aroma and flavor are very good. They don’t get contaminated with the smell of soil, rubber, tobacco, mold, or uncontrolled fermenting fungi” (BL1-5, 2023). Tiến avoids yeast-inoculated processing because it is hard to manage: “The global trend now is to add artificial yeast to create coffee flavor, but that can be very dangerous [to coffee quality] if not controlled properly. The yeast can multiply at a very alarming rate” (BL1-6, 2024). Here again fermentation presents both possibilities for differentiation and risks; processors and hired workers labor between the two through their embodied knowledge. Producers know they do not labor alone and it is partially their knowledge of this collectivity that drives the physio-cultural realization of value and qualities (Besky and Blanchette 2019).

#### Sharing fermentation

Producers also build knowledge in social and commercial networks, as competitors are also neighbors, allies and sources of expertise and insight. Producers often exchange information at social gatherings in private or public spaces like cafes. They make informal farm or mill visits, engage in discussion via social media groups (Zalo and Facebook) and attend events like cooperative meetings, industry gatherings, cultural festivals and weddings. By exchanging in-depth knowledge of technique (*know-how*) and markets (*know-what*), they learn how both practices and value chains enable quality. This helps them produce coffee that is not just “different” but “better” (Fischer 2022, p. 57).

Processors are allies in pushing to build markets for Vietnamese specialty coffee, domestically and internationally, and they celebrate each other’s successes. One place this happens is at “new crop” events after harvest season, often hosted by roasters. These involve public cuppings of coffees from many producers, sometimes as part of a contest with judges. Producers discuss challenges and stories from the harvest and get to taste their competition.

At one such event in 2024, cupping revealed one lot from a well-respected producer, Trí, that did not match expectations: judges and attendees agreed something had gone amiss during fermentation. Some noted it tasted phenolic—of a class of compound which, when abundant or in the wrong ratios, tastes medicinal or woody (Cardoso et al. 2021). In the competition results, this lot scored in the low 60s while the other seventeen lots nearly all scored between 81 and 85.

But Trí said he guessed this coffee would score poorly and shared it anyway as it was an experiment. He explained to the gathering what might have gone wrong: the coffee was natural processed, inoculated with purchased yeast from the labs of a multinational supplier and fermented for 48 h. He suspected the yeast had worked quicker than anticipated and consumed too much of the available sugar, becoming stressed and then producing unwelcome, “deep fermented” notes.

Processors like Trí and Tiến understand the principles of fermentation and distill technical scientific concepts into actionable practices. They use cherries at optimal ripeness, feeding bacteria and yeast requisite polysaccharides, nurturing them in a hygienic environment, facilitating their production of enzymes (ex. pectin lyases) that depolymerize and hydrolyze sugary fruit (Silva 2014, 402–422). However they do not know their coffee’s chemical constituents. Ratios of, say, 2-methylpyrazine (nuts, chocolate) to 4-vinyl guaiacol (smoky, spicy) and beta-damascenone (fruity, honey) are essential to the production of value (Pinheiro et al. 2021) but to the culture of production, they remain unarticulated. As processors experience, experiment and create, they establish the bounds of what they need to know.

Trí’s sharing prompted a discussion among those present about the messiness of microbial management, aspirations of quality and the limits of what they can know without a microbiology lab. Similar to natural wine producers in Anna Krzywoszynska’s work (2015), many in these processors and their networks realize one of the qualities of their coffee *is* uncertainty, at least until roasted and cupped. Further, the shifting potential of microbial metabolisms for Vietnamese coffee plays out in this lot of coffee: fermentation is a biological engine of possibilities, of both value and loss.

#### Reading fermentation

But for Tiến and other early producers of specialty coffee in Vietnam, there was no local network and they turned to online networks for new ideas. Tiến’s family comes from northern Vietnam and after moving to Lâm Đồng and growing commercial coffee for a few years, he began teaching himself the basics of producing better coffee from YouTube, supplementing it with written sources. He got help from friends to translate information from English (guides on processing, industry publications). He then adapted these to his materials, needs and context. Today, the processors involved in this research constantly draw on information from online sources, from coffee industry publications to blogs and social media posts.

A minority of specialty producers (*n* = 7, of 54 interviewed) also consult scientific research publications and share this information with their networks. Nearly all research they consult is in English and most get translation help from friends, Google or ChatGPT. Those with better English skills have far better access. One younger processor, conversational in English, voiced skepticism about Vietnamese translations of coffee reference books: “I searched all Vietnamese materials, but I couldn’t find accurate knowledge compared to what I know form in-depth learning” (DL8-1, 2024). There are two recent Vietnamese books that help to address this gap: *Khoa Học Cà Phê* (“Coffee Science”; Nguyễn 2022) and *Lên Men Cà Phê* (“Coffee Fermentation”; Nguyễn 2024). The books translate information already widely available in English books and papers for a broad Vietnamese audience, from farmers and processors to consumers. Projects like this, to make expertise more accessible, echo longstanding calls for “democratization of science” in agriculture (Carolan 2006). Where agricultural producers seek to break from local norms but lack reliable knowledge networks, such projects are critical.

#### Studying fermentation

For some coffee producers, formal taught learning (courses, workshops) has helped translate evidence or principles like the above into practical recommendations. Some specialty producers joined classes with specialty coffee trainers or, less commonly, workshops (hội thảo) offered by agricultural or coffee export companies. The latter address issues like pest management or more environmentally-minded cultivation, often with an emphasis on the products the company sells; producers of all grades of coffee expressed skepticism of these. However such workshops are also often supported by public-private collaborations, meaning they have funding for agricultural engineers and extension workers to work directly with producers.

For specialty coffee, there are formal courses such as the CQI’s Q Processing series which covers fermentation. These are often taught by foreign experts from coffee producing countries, however there are now a few Vietnamese instructors. Producers also stand to benefit from Q Grader courses, as these let them participate directly in categorization processes (more on category construction below). However the high fees and time requirements for Q courses (normally six full days) also make them inaccessible for most producers. In early 2025, a Q Grader course in Vietnam cost over 2,300 USD, comparable to costs in Europe. Producers who joined courses were financially successful, sponsored by benefactors (ex. roasters) or received discounted tuition. This put some producers into relationships of patronage with other industry actors. Overall, whether and how Q Grader courses were “worth it” for producers remained an open debate.

One recent attempt to make this knowledge accessible to more producers was a series of processing trainings organized by a large Vietnamese state-owned coffee company, an international development organization and two coffee cooperatives who supplied the company. Taught by one foreign and one Vietnamese expert, the trainings aimed to help farmers and processors increase their knowledge of processing and fermentation techniques. The producers are then free to sell their coffee, hopefully improved, to whoever they choose. Specialty-specific training within a larger-scale value chain suggests shifts in flows and functions of knowledge, as lead firms and development discourses make fermentation expertise part of modern, “sustainable” coffee production. Though these first trainings attracted as many company employees as farmers or processors, other emerging fora, like the Vietnam Amazing Cup green coffee competition, also see lead firms, industry associations and the state (primarily provincial and local government) endorse versions of specialty coffee as a path to development.

The need to make training more accessible is part of broader efforts in Vietnam’s specialty coffee networks to share knowledge in order to scale up production and build shared understanding—to “democratize knowledge” in specialty coffee, as one roaster and coffee consultant put it (SG1, 2024).

This is already the work of commodity producers. Though it looks very different for processors like Huy, Trí or Tiến and hired workers, for instance, they all sift through technical, experiential, local, expert, lay, embodied and other knowledges—sorting the wheat from the chaff, so to speak—to enact a working assemblage of knowledges.

#### Fermentation knowledge in action

On a hillside in Lâm Đồng, a producer named Mai and her family run a Robusta mill that illustrates how the ways of knowing fermentation discussed above operate at a larger scale. Their three-hectare plot is split between processing facilities—outdoor wet mill, indoor dry mill, drying yards, warehouse, drying greenhouses, mechanical dryer—and around 1.5 ha of Robusta trees that Mai’s parents first planted in 1987, early in Vietnam’s coffee boom.

On a visit in 2024, she talks me through today’s fermentation protocol while I sit cutting up a dozen pineapples outside her parents’ home. Beside us are thirty-odd blue plastic barrels. They hold thick, wet masses of honey-processed coffee mixed with already decomposing chunks of pineapple and untold intermingled communities of industrious bacteria and yeasts.

**Fig. 5:**
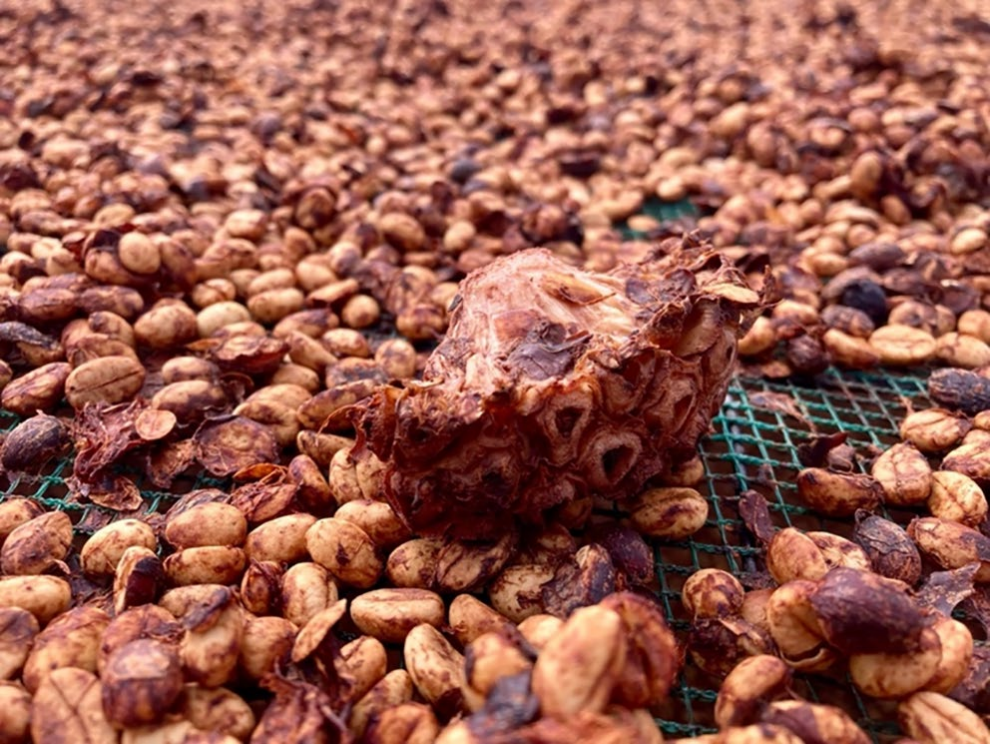
Honey-processed Robusta coffee, fermented with pineapple, dries

Today’s cherries will be cleaned, sorted and put into plastic bags for seven days, their pH checked daily by digital meter. They are then pulped, mixed with pineapple and fermented for at least two more days before drying (Fig. 5). When we stir the slurry in the barrels, the smell of souring fruit is heady. The pineapple provides sugars, enzymes and microbiota but when the coffee is hulled and roasted, it smells nothing like pineapple. One roaster says it develops warm spice and lightly floral notes. These notes far exceed what the market expects: the use of any flavor notes at all on retail bags of Vietnamese coffee or Robusta was unheard of until the mid-2010s; it is still rare outside the country.

Mai built her expertise through networks of roasters, farmers and other processors. After attending university in Ho Chi Minh City, in 2012 she returned to work with the family’s processing and trading business. In 2014, she saw the nascent market for differentiated coffee in Vietnam and began working to increase the quality of a portion of their production. Mai focused on purchasing ripe cherries from other farmers and processed them carefully. In 2015, she joined one of the first processing trainings in Vietnam taught by an internationally-renowned expert, which connected her to crucial knowledge networks.

She also built relationships with enterprising local farmers, roasters and exporters. Some roasters partnered with the mill to produce both lower-cost lots and experimental fermentations, allowing Mai and her family to scale up higher quality production. In 2023, Mai’s family, over 300 farmers and two roasteries formed a cooperative, giving them access to some limited government support. Cooperatives in Vietnam have historically taught farmers who migrated to the Central Highlands about coffee cultivation, in Vietnam’s era of collectivized agriculture (1976-86) and the early post-Đổi Mới years. Most came from lowland areas and had to learn to grow coffee, often from one another.

Mai’s new cooperative and its partnerships return to those collective efforts and challenges, but shift the focus to commodity differentiation and knowledge of high-quality coffee production, including processing. The cooperative built processing facilities separate from Mai’s family business, allowing other farmers more agency and autonomy. As of 2024 however, the family business was still the primary economic actor and the prospects for more widely distributed benefits were still unclear. The cooperative’s success depends on whether it can produce commodities that are legible within architectures of specialty coffee quality.

### Knowing categories: fermenting value

Producers’ knowledge creates value by contributing to the construction of legible commodity categories, with their role and agency reflecting the relationship between their knowledge and value chains. Categories group characteristics, allowing buyers to register and value certain qualities. Coffee buyers may seek a specific flavor profile, origin, price point or fermentation method. One roaster in the UK prefaced our conversation about Vietnamese coffee samples by saying his business focused on purchasing natural coffee (whole cherry fermentation and drying), with fruity notes and full body (UK5, 2025). Other buyers were most interested in fermentations that add complexity to aroma and flavor for the sometimes-unremarkable Catimor variety. For coffee blends, buyers often need to ensure the commodity is dependable, with mass market brands potentially containing dozens of coffees. They seek sweetness, acidity or body that is replaceable. This use of categories in blending of commodities echoes Sarah Besky’s analysis of blending for tea (2020).

Coffees like Mai’s, Trí’s, or Tiến’s can only be differentiated and valued as legible categories take form. When a particular ordering of processing practices becomes regular and solidifies, processors assign a label for the processing protocol (phương pháp sơ chế). The processing method thus forms a category and the commodity now qualifies as “extended fermentation honey Robusta”. The coffee both meets and makes the conventions of the category.

This is the work of differentiation, in which commodity producers’ livelihoods depend on *know-what*: practical knowledge of a commodity’s possibilities. One second-generation producer in Đức Trọng district of Lâm Đồng described his introduction to the category of specialty coffee:

Honestly, when I first started, I didn’t even have that concept. I didn’t know what specialty or high quality meant. I just had green beans, brought them to different people for roasting and listened to their evaluations. Only later did I really understand what I was doing… I learned from the community… roasters, farms, a few people… Like, Duy [a roaster] could hold the green beans, smell them and immediately know what the issue was… what errors there were, so I could adjust my processing (LD20-2/3/4, 2024).

This producer adapts practices to fit his context: his farm (3 ha Robusta) has no electricity for a mill and so he uses only natural processing, drying cherries whole. He has still partnered with two Vietnamese specialty roasters on special fermentation methods, with the roasters providing yeast, developing a protocol in cooperation with him and committing to buy the entire lot—only 100 kg in total, but at over three times the price of commercial green beans. Though his revenue from those lots is minimal, they help him build relationships and the volume is increasing.

For roasters, processing information is critical. By featuring processing methods and other production information in marketing, they capture a large portion of the value produced in the labor of careful fermentations. Processing knowledge is also crucial for roasters’ physical work as it affects how beans behave in roasting, packaging and brewing. Along with cupping, information on fermentation helps roasters understand how a coffee fits or disrupts categories. Producers show their coffee is reliable—that their beans carry desirable precursor compounds, rather than ochratoxins. Tiến’s use of steel tanks and air-conditioned rooms, for instance, signals his coffee is clean and will likely meet specialty buyers’ exacting standards. He signals that he has harnessed fermentation for quality, and that his coffee belongs to a category free from microbial liabilities.

Despite this, some roasters and processors avoid the term “over-fermented”, for its normative connotations; in the words of one of Vietnam’s first specialty roasters, “There is only unexpected fermentation” (SG3-1, 2024). However in practice, people throughout the value chain diagnose when yeasts and bacteria produce objectionable cup characteristics. As one Robusta producer put it, “Basically, don’t let it go ‘over’, like that pickled smell, or like cucumbers left too long. So you have to smell it regularly” (LD1-1, 2023). One SCA trainer and grader who works throughout Asia described how the “funky”, spicy notes now common in good Vietnamese Robusta reflect particular ideas spreading among Vietnamese producers and consumers about “how flavor should be”. Those conceptions affect which fermentation practices are shared and taught (SG9, 2024). Here, social knowledges of the working ecosystems on farms and in mills (Besky and Blanchette 2019) become a bridge between the mechanics of fermentation and the mechanics of markets. Managing fermentations and expectations go hand-in-hand.

Information shapes categories by defining the good for exchange, making material attributes legible. As Besky writes of tea and its criteria in India, “To tell the story of quality is to explore historically particular ways of relating to the material world through knowledge (both linguistic and embodied) and work (both productive and reproductive)” (Besky 2020, p. 5). Qualities become consequential when they establish categories, facilitating valuation (Liberman 2022).

To function, categories require standards, which McDonell argues are essential to materializing concepts of quality (McDonell 2023). The impacts of yeasts and bacteria register in value chains when (or if) they become legible within emerging standards. While most buyers follow globalized norms such as the SCA/CQI system, some deviate: some Vietnamese roasters value distinct funky or winey flavors, in Robusta or Arabica, which are less desirable for some export markets.

As producers learn particular buyers’ standards, this knowledge allows them to create value. For commercial coffee, farmers may be able to access a slightly higher price if they understand the standards local buyers use to set prices, like a higher percentage of ripe cherries, fewer defects and less foreign matter. These buyers may be traders or processors. Some farmers understand that standards of cleanliness and ripeness save labor downstream, as the coffee requires less sorting. Though this knowledge is not essential to their labor (Taylor and Bhasme 2025), farmers use this *know-what* to attempt to negotiate value.

The construction of categories thus splits an undifferentiated commodity like “coffee” into multiple commodities (“coffees”) identified by distinctive characteristics, whether notes of stone fruit and flowers, bitterness or a percentage of fungus-damaged beans. Whether driven by processors or downstream buyers, shifts in fermentation practices can open up spaces of ambiguity in moments of exchange and consumption. Though it may not affect power relations with value chains, this cultural negotiation also introduces blurriness and uncertainty into categories. Drawing on Michel Callon’s work, Anna Krzywoszynska suggests that for some wine, producers can push buyers towards a “taste for uncertainty”: to value not just standardization but also diversity and difference (Krzywoszynska 2015, p. 496). This potential for reflexivity is central to the work of Mai and her partners, as fermentations help shape new categories.

But as Krzywoszynska notes for wine, most value chains seek to resolve uncertainty and there are limits to the benefits of novel fermentations. Buyers require that supplies and their qualities be stable. For producers, experimentation and creativity can risk livelihoods and relationships. Coffee roasters frequently ask processors for particular fermentations, sometimes committing to purchase whatever coffee results but often not. Barring exceptions, producers bear costs and risks as the market pushes them towards trendy practices and ever more herculean processes.

Vietnam’s coffee industry also faces the project of building new categories for Robusta: showing how Robusta, when treated well, can be as refined as Arabica. Though still distinctly its own entity, Robusta therefore deserves to move, along with its farmers and processors, within networks of connoisseurship. At Mai’s mill, for example, workers and family carry out a range of processes and experimental fermentations that push the edges of what Robusta can be. All roasters consulted in the UK (*n* = 8) said they do not buy Robusta, though they expressed interest in understanding specialty Robusta as a category. Specialty Robusta is slightly more common in the USA and elsewhere in Europe, due in part to the Vietnamese diaspora, however it remains a rare alternative.

Specialty-grade Robusta requires new categories, exceeds standards and expectations. Uyên, owner of a large specialty processing-roasting business in Đắk Lắk, described it as follows:

[International buyers] just drink and know about commercial Robusta, so they think that Vietnam only produces coffee of the lowest quality. So Vietnam has no voice. But now it’s changing because we are doing better quality, and we bring it to the world, to introduce everyone to how we make coffee, the taste of fine Robusta, and they can understand that, okay, Robusta is not bad… [It is] not the instant coffee, not only really bitter… Internationally, they know how to make good coffee, right? But they don’t have Robusta. They just have Arabica. So they don’t exactly know how Robusta truly is (BMT-8-3,4, 2024).

Similar to Mai, Uyên’s work is about testing conceptions and perceptions of what Robusta can be. Her roastery markets a line of “experimental” process coffees, though these are almost exclusively for the domestic market—the category of local wine yeast-fermented Robusta, for instance, has only recently coalesced within Vietnam and the mill involved chooses not to produce enough of this coffee for export. The primary impact on the value chain from experimental coffees is to contribute to the construction of new categories, including a diversity of fine Robustas that open up possibilities for novel conventions.

As Uyên put it: “In Vietnam five years ago, some specialty coffee businesses focus on Arabica, never thinking about Robusta. But with us, we think Robusta has a different way, a different taste.” This Robusta advocacy within the value chain is potentially one of the most impactful aspects of specialty coffee in Vietnam. Uyên continues: “Each has its own delicious taste. Arabica is delicious as Arabica, Robusta delicious as Robusta (*Mỗi thứ có một cái ngon riêng. Arabica ngon Arabica*,* Robusta ngon Robusta*)” (BMT8-1, 2024). But Robusta and its producers face major challenges in getting structures of qualification, including Q grading, to register its qualities.

Prospects for Robusta are very different in Vietnam’s domestic market, where established cultural preferences for Robusta mingle with imported architectures of quality and value. Vietnamese coffee culture is already built upon Robusta’s common material strengths: notes of dark chocolates, vanillas, nuttiness and rich body. These are essential for now-traditional Vietnamese drinks like cà phê sữa đá (iced, with condensed milk). Vietnamese roasters have long added roast corn, sugar, fish sauce, rice alcohol, flavorings and other additives or bulking agents that played up these same strengths. There have been high-profile scandals in which companies or roasters were caught adding chemicals from batteries to change the coffee’s color and save on costs. As a result, differentiated coffee in Vietnam began with simple propositions of *pure* coffee (cà phê nguyên chất). From there, more ambiguous categories like cà phê sạch (clean) and cà phê bền vững (sustainable; see Grant 2022) emerged.

As specialty coffee has taken off in Vietnam, specialty producers, domestic roasters and their discerning consumers have built categories and preferences that reflect but also depart from international conceptions of quality, especially for Robusta. Specialty Robusta and its diverse qualities are more desirable and valued within Vietnam. Vietnamese knowledge of Robusta production and consumption stands to influence global architectures of quality within the SCA and beyond, though this is only just beginning. Vietnamese producers already know the “cái ngon riêng” of Robusta and this knowledge introduces new possibilities for value chains, especially through roasters in Vietnam. New possibilities for tastes, flavors and their perception depend on harnessing communication at each point along the chain as coffee moves to consumption.

## Discussion and conclusion

The production of value and qualities via fermentation, from mills to fermentation tanks, to cuppings and evaluation, demonstrate how coffee producers’ livelihoods depend on flows of not only coffee and its qualities, but also of knowledge. By tracing functions of knowledge throughout the value chain (Fig. 2), this paper shows how a commodity producer’s agency reflects the ways their knowledge and knowledge systems fit within, disrupt and enable value chains. Producers assert agency by using their information about production, including fermentation, to attempt to build new categories. Global value chains, for coffee and beyond, are grounded in plural possibilities of multiple types of quality and value (Krzywoszynska 2015, p. 496) and “contaminated diversity” (Tsing 2015, p. 33). Transposed for other commodities and places, the framework of working knowledge allows cultural economy and value chain studies to better understand the positions and experiences of commodity producers.

By centering producers’ decisions, the approach allows more careful analysis of their constrained agency in social-ecological networks—of the contingent and uneven relationships that bring coffee, its qualities and its values into being, and into cups. Legible categories carry coffee through mills, warehouses, quality checks, cargo holds, roasting drums, cooling trays, grinders and brewing apparata. But categories also reflect perceived binaries of expert/lay, specific/general and local/formal knowledge (Carolan 2006). They also enact a hierarchy of epistemic authority (Zinsli 2023), in which definitions of coffee quality are subject to both capital and culture.

The contingencies and the experimental nature of quality (Besky 2020) offer avenues for understanding producer agency in value chains, even if exploitative distributions of power and value remain largely unchanged. Specialty processors in Vietnam are beginning to impact the global industry by bringing their coffee and knowledge of its production into markets. Their coffee shapes new market categories that anonymized, commercial-grade Vietnamese coffee never required. One Robusta producer described his coffees’ impact on markets:

The goal is always to try to stay ahead—to lead, to be the leader in high-quality products and move toward higher-end customers. If I succeed, many farmers will follow, just like when I first started, there was no one, but after I did it, now hundreds of Vietnamese farmers are following this path (BL1-8, 2024).

As diverse commodities and the knowledges they embody contribute to categories, they introduce possibilities for new ways of working. This suggests at least two paths forward for research bringing together global value chain and agrarian studies, reflecting the two acts of translation discussed above: first, research can develop richer understandings of producers’ livelihoods by accounting for how they assemble diverse knowledges via social-ecological networks—the networks that bring coffee off trees and into hands, baskets, bags, pulpers, fermentation baths and hulling machines. Tracing knowledges can unpack why production looks and functions as it does. Second, studies of global value chains and livelihoods can present a fuller view of the production of value(s) and quality(ies) by mapping who constructs categories for commodification, how and to what end.

More broadly, this paper thus argues for a political-ecological analysis of all human work as knowledge work. Studies that foreground specific producer knowledge (be it situated, indigenous, embodied, working or otherwise) are even more essential to political economy than previously thought precisely because they are integral to producing value. In the Vietnamese context, there is a need for ethnographic studies of localized expertise to document specific functions of producer knowledge—building on work by, for example, Ha T. N. Huynh (Huynh et al. 2022) and Mai Phuong Nguyen (M. P. Nguyen et al. 2020). Careful, contextual analyses of actor knowledge can push studies of cultural economy and commodities to better understand producers’ agencies amidst the inequalities of value chains (Lang et al. 2023).

The functions of producers’ working knowledge vary depending on their relations and positions in the value chain. In the domestic market for Vietnamese specialty coffee, producers can more easily build reputations for themselves and their coffees, when feedback loops are short, relationships are close and Robusta is recognized. But in the global industry, norms and conventions reflect hierarchies of knowledge systems: Vietnamese coffee, and Robusta or *canephora* more broadly, constitute disruptive commodities. In fermentation and production that disrupts categories, there are possibilities of new conventions for the unconventional.

For some Vietnamese coffee producers, there is an opening in the uncertainty and frictions of categorization, and more specifically in what Krzywoszynska calls “open taste” (2015, 500): the commodification of materials based on novel, lively qualities that might shift expectations. Experiences and knowledge of coffee’s lively possibilities vary among farmers, processors and hired workers; fermentation may be a threat, a tool, an opening to new markets, or incidental. An analysis of working knowledge and its function within value chains can speak to producers’ agencies and constraints—and the limits on and possibilities for making a better living.

## Data Availability

Not applicable.

## Footnotes

1 All names and identifying details have been changed.

2 Robusta is a variety of *Coffea canephora*, the other most common variety being Brazil’s Conilon. In this paper and much of the industry, “Robusta” refers to Canephora generally, despite the inaccuracy. Canephora represents 43% of global production; the rest is primarily *Coffea arabica* (Lewis 2025). Canephora has much higher yields than Arabica per area, often twice the caffeine, is more resistant to pests and diseases, grows at lower elevations and is more adaptable to climate change.

3 The SCA is global but was formed when predecessor associations for the USA and Europe merged in 2017.

4 This research was conducted before the SCA introduced the new Coffee Value Assessment tool (late 2024) and took over the Q Grader program (2025).

5 There is extensive interdisciplinary literature focused on coffee botany, agronomy, microbiology, sensory science and much more. *Coffee Science* is a popular open-access journal.
